# Developing the Metacognitive Awareness of Grit Scale for a better understanding of learners of English as a foreign language

**DOI:** 10.3389/fpsyg.2023.1141214

**Published:** 2023-04-11

**Authors:** Mingzhe Wang, Lawrence Jun Zhang, Richard Hamilton

**Affiliations:** Faculty of Education and Social Work, University of Auckland, Auckland, New Zealand

**Keywords:** metacognition, grit, instrument design, CFA, EFL, MCAGS

## Abstract

The prominent impact of metacognition on learners’ academic achievement is widely discussed. Learners armed with appropriate metacognitive strategies should witness enhancement in learning performance. Similarly, the concept of grit is also valued as a crucial factor contributing to the improvement of academic achievement. Nevertheless, discussion of the relationship between metacognition and grit or their collective influence on other educational and psychological variables is limited, not to mention that an instrument measuring learners’ metacognitive awareness of grit is a desideratum. Hence, by incorporating the constructs of metacognition and grit, the present research developed a measurement scale to address this need, named the Metacognitive Awareness of Grit Scale (MCAGS). The MCAGS consists of four components and initially included 48 items. It was later distributed to 859 participants for the purpose of scale validation. Confirmatory factor analysis was applied to evaluate the scale’s validity and explore the factor-item relationship. A final model containing 17 items was retained. Implications and future directions were discussed.

## 1. Introduction

With more than four decades of research, metacognition is identified as a crucial aspect of our working and learning experiences. It is often defined as one’s awareness and regulation of their own knowledge, experience, and emotions (Zhang and Zhang, 2018, 2019; Rivers, 2021; Sun et al., 2021; Sun and Zhang, 2022). As an underlying psychological process that constantly coexists with our cognitive activities, it is also closely connected with other research realms, including educational and positive psychology (e.g., Stanton et al., 2021; Wang and MacIntyre, 2021). Applying metacognitive strategies while learning improves the learning experience and achievements (Stanton et al., 2021). Mastering the methods to evaluate and manage the learning process is vital to becoming an effective and successful learner. Scholars also share such consensus with interest in exploring the English as a Foreign Language (EFL) learning experience. Research has pointed out that students armed with pertinent metacognitive knowledge and strategies when learning English often witness significant enhancement in academic performance (e.g., Zhang and Zhang, 2019; Teng, 2020) and self-efficacy (e.g., Chen and Zhang, 2019; Shehzad et al., 2020; Chen et al., 2021).

Apart from the interplay between metacognition and EFL, scholars have recently drawn inspiration from the research realm of positive psychology and integrated it into the EFL learning research (MacIntyre and Gregersen, 2012; Liu et al., 2022; Zhang et al., 2022). Findings from the last decade have revealed that positive emotions often play significant and positive roles in EFL learning (e.g., Jiang and Dewaele, 2019; Wang, 2021). Wang et al. (2021) proposed a model containing seven factors that may contribute to EFL learners’ learning experience: grit, resilience, well-being, emotion regulation, engagement, loving pedagogy, and foreign language enjoyment. Most researchers in these areas argue that individuals who possess high levels of these qualities exhibit stronger tendencies to engage in learning activities (e.g., Jin and Zhang, 2019; Mercer and Dörnyei, 2020), a high capacity to adapt to different or unfavorable circumstances to enhance learning motivation (Kim and Kim, 2021), and constant interest and effort in language learning activities (Khajavy et al., 2021). Nevertheless, despite the fruitfulness of positive psychology research, the positive psychology perspective of EFL research is still in its nascent state. One promising avenue for future research is to explore the relationship between positive psychology and language learning achievement from a metacognitive view. A review by Wang (2021) indicates that self-regulated learning strategies guided by metacognition may assist learners’ perseverance of effort (grit) in learning. Conversely, grit may also result in persistent self-regulation. The present research also seeks to connect grit, metacognition, and EFL.

Due to the nature of metacognitive research, which probes into people’s thoughts about their own thoughts, one is not able to observe or measure it directly. In this research domain, most researchers applied a self-report questionnaire method. Hence, various instruments were created to measure individuals’ general metacognitive awareness and related psychological variables. However, no existing instruments were created to directly connect grit with metacognition and measure to what extent English learners will evaluate and regulate their knowledge and strategies for maintaining or improving their grit levels. In the present research, we sought to extend the research area by developing a new instrument assessing individuals’ metacognitive awareness of grit within the EFL context, namely the Metacognitive Awareness of Grit Scale (MCAGS). Confirmatory factor analysis (CFA) was performed to evaluate the scale validity and eliminate defective items. In the section which follows, we discuss the concept of metacognition, positive psychology, and grit, followed by reviewing the recent literature and introducing the existing psychological measurements.

## 2. Literature review

### 2.1. Metacognition

The notion of metacognition encompasses two domains (*metacognitive knowledge* and *metacognitive regulation*), according to Flavell (1979, p. 907). *Metacognitive knowledge* (or *metacognitive awareness*) implies individuals’ knowledge regarding themselves or others as cognitive processors. The knowledge or beliefs about one’s own capabilities to acquire desirable results or to what extent one’s characteristics, such as gender, age, and personality, can have consequences on learning processes (Stanton et al., 2021). In particular, learners’ awareness of their identities as learners, the requirement of the current task, the strategies to exploit (*Declarative knowledge*), how to use strategies (*Procedural knowledge*), and when and why to use them (*Conditional knowledge*).

Research revolving around metacognitive awareness commonly focuses on (a) assessing individuals’ metacognitive awareness, (b) relating metacognitive awareness with other influential factors in learning, (c) exploring what learning strategies are favorable, and (d) promoting metacognitive awareness. Empirical studies have focused on the interactions between metacognitive awareness and other variables. For instance, learning achievements and performance are the influential factors that were given a significant amount of attention, such as the impact of metacognitive awareness on learning achievement for specific subjects (e.g., Mathematics learning; Bulut, 2021; foreign language learning; Mäkipää et al., 2021), for disparate groups of learners (e.g., undergraduate students; Pradhan and Das, 2021; secondary school students; Jaleel, 2016), and the interplay between metacognitive awareness, learning achievement, and other psychological variables (e.g., emotions; Wang and MacIntyre, 2021; motivation; Cakir and Guven, 2019). In light of the influence of metacognitive awareness on learning achievement, researchers have also been evaluating potential ways to promote learners’ metacognitive awareness, such as problem-based learning (Kuvac and Koc, 2019), online flipped classrooms model (Khodaei et al., 2022), and collaborative intervention (Sandi-Urena et al., 2011). Finally, as stated in the above section, scholars in this research domain have primarily used questionnaires to assess individuals’ metacognitive awareness indirectly. Diverse instruments were developed to measure general or domain-specific metacognitive awareness, such as the Metacognitive Awareness Inventory (MAI; Schraw and Dennison, 1994) and the Strategy Inventory of Language Learning (Oxford, 1990). We will discuss this later in greater detail.

Another crucial aspect of metacognition is *metacognitive regulation* (or *metacognitive experiences*; Flavell, 1979, p. 906), which describes the regulation or adjustment of learners’ learning experiences that help them control their learning. Knowing the existence of learning strategies is not sufficient. Learners also need to exploit them in their learning actively. *Metacognitive regulation* embodies three manifestations: *Planning*, *Monitoring*, and *Evaluating* (Stanton et al., 2021). Each of these three components represents a phase of our learning process, from *planning* the appropriate strategies we will apply in response to a new task to *monitoring* the effectiveness of the selected strategies and finally arriving at *evaluating* the current plan and adjusting it for future tasks and better performance. Hence, scholars have also invested much energy in exploring their roles in learning. For instance, the vast number of studies investigating practical metacognitive strategies involve presenting novel information to aid learners’ *planning* process. A mixed-methods study by Zhang et al. (2021) reported that EFL learners do not actively apply metacognitive strategies (specifically, the problem-solving strategy) when performing listening tasks, and the use of strategies largely depends on task difficulty. They suggest that such findings underline the necessity to emphasize and encourage the selection and use of metacognitive strategies when learning. At the same time, monitoring and evaluating strategies are also crucial for a successful learning experience, thus attracting much attention from scholars. Past research elucidated that learners’ monitoring accuracy can significantly influence overall test performance (Thiede et al., 2003), while monitoring accuracy can also be improved through learning strategy instruction intervention (Gutierrez de Blume, 2022). After a test, learners can also apply *evaluation* techniques to reflect upon the effectiveness of the strategies used when preparing or during the test (Stanton et al., 2021).

#### 2.1.1. Metacognition in EFL contexts

Working within Flavell’s framework (Flavell, 1979, p. 907), scholars in the EFL learning research realm primarily incorporated metacognition within specific domains or skill areas of EFL learning, for example, reading, writing, listening, speaking, vocabulary, and grammar. Most early empirical and theoretical research on metacognition focused on second-language reading (Zhang and Zhang, 2018). The qualitative research conducted by Zhang (2010) are apt examples. In his work, the disparity of Chinese EFL students’ metacognitive knowledge was found to be strongly influence their EFL reading comprehension. In a similar vein, Dabarera et al. (2014) revealed a relationship between metacognitive awareness-raising and reading comprehension improvement in their mixed-methods research. It was not until the early 1990s that researchers realized the complexity of second-language writing and began to appreciate the importance of metacognitive writing awareness and strategies (You and Joe, 2001). For instance, EFL writers’ motivational beliefs and self-efficacy were found to be strong predictors of English writing self-regulated learning strategies (Teng and Zhang, 2020). A study by Sarbazi et al. (2021) reported that vocabulary, syntax, and learners’ metacognitive reading strategies could collectively predict changes in English reading comprehension. Moreover, a study on listening for EFL learners unveils that metacognitive intervention in their first language significantly improved their EFL listening performance (Bozorgian et al., 2021).

Evidently, studies revolving around metacognition in relation to the learning and teaching of EFL do show the importance of the Flavell (1979, p. 907) framework. The framework, along with its sub-components, is a crucial theoretical construct not only for EFL researchers but also for the development of metacognitive measurement tools, such as the MAI (Schraw and Dennison, 1994). Hence, such a framework also guides the present research on developing the Metacognitive Awareness of Grit Scale in the EFL context.

### 2.2. Positive psychology

Martin Seligman brought positive psychology into the field of psychology in 1998 (Seligman and Csikszentmihalyi, 2014) as a response to past practices that focused on maladaptive behavior and thinking for underlining the importance of exploring factors that contribute to happiness and well-being. It is bolstered by the *broaden-and-build theory*, which underscores that positive emotions cultivate broadened mindsets and creativity (Fredrickson, 2004). For years, researchers have primarily been attracted by the idea of negative factors that could have detrimental effects on English language learners’ motivation and achievement, such as boredom (Li et al., 2021), anxiety (Su, 2022), and burnout (Li et al., 2021). Nevertheless, inspired by positive psychology, academia in this field is shifting in interest, arguing that instructing individuals to avoid negative emotions is insufficient. We should encourage ourselves to pursue eudemonic well-being and resolve obstacles from positive perspectives (Jin et al., 2021). Despite the appreciation of the prominence of positive psychology in the field of second language acquisition from early researchers (e.g., Arnold and Fonseca, 2007), the expansion of such a research interest began after MacIntyre and Gregersen (2012). More recently, as mentioned above, Wang et al. (2021) proposed a model containing seven factors from positive psychology that significantly impact English language learning and teaching and suggest promising future research avenues. Among them, the concept of *grit* has caught our attention.

#### 2.2.1. Grit

Duckworth et al. (2007) initially defined the notion of grit as “perseverance and passion for long-term goals” which differentiates it from resilience and self-control. Although found to be correlated with grit (Credé et al., 2017), both resilience and self-control lack long-term commitment characteristics. Gritty individuals remain committed to their goals and exhibit resistance to the impact of setbacks (Duckworth and Gross, 2014). Grit embodies two dimensions: *consistency of interest* (COI) and *perseverance of effort* (POE). The concept of COI describes the ability to maintain consistent interest in an activity despite failure and obstacles. At the same time, the idea of POE refers to the steadfast pursuit of targets and the ability to exert hard work when confronting hardships. This individual difference variable is often perceived as an influential factor contributing to the distinct performance of people with similar levels of cognitive ability (Wei et al., 2020). In light of the malleable nature of grit, enhancement through intervention and instruction is possible (Clark and Malecki, 2019). Teachers in educational institutions can take advantage of such traits to train and enhance students’ grit levels to improve their learning behaviors.

Much research has been conducted to explore the relationship between grit and academic achievement (e.g., Credé et al., 2017). Since successful second language learning depends on consistent effort, the connection between grit and learning achievement grabbed the attention of scholars in Second Language Acquisition. Teimouri et al. (2020) explored the relationship between EFL learners’ grit and language achievement using the language-specific grit scale and revealed that L2 grit is positively related to language learning motivation and achievement. Furthermore, Sudina and Plonsky (2021) even revealed that the two dimensions of grit exert different influences on language achievement, with the consistency of interest serving as a more potent predictor of achievement than the perseverance of effort. Aside from the direct impact on achievement, grit was also found to be correlated with other factors that indirectly or collectively affect language learners’ performance. For instance, Changlek and Palanukulwong (2015) reported that motivation is positively related to grit, while anxiety exhibits an inverse relationship. A study by Liu and Wang (2021) found that grit is positively related to foreign language enjoyment while negatively correlated with foreign language anxiety. Foreign language enjoyment also serves as a potent mediator between the effect of grit on language achievement.

### 2.3. Psychological measurement

As mentioned previously, the original instrument measuring learners’ general metacognitive awareness and experience was developed by Schraw and Dennison (1994), named the Metacognitive Awareness Inventory (MAI). The construct of MAI entails two levels that follow the metacognitive framework proposed by Flavell (1979): *knowledge of cognition* and *regulation of cognition*. According to work by Schraw and Dennison (1994), *knowledge* about our cognition consists of three subprocesses: declarative knowledge, procedural knowledge, and conditional knowledge. The *regulation* of our cognition includes five component consisting of planning, information management strategies, comprehension monitoring, debugging strategies, and evaluation (Artz and Armour-Thomas, 1992). Example items from the MAI are “I understand my intellectual strengths and weaknesses” (declarative knowledge), “I set specific goals before I begin a task” (planning), and “I focus on the meaning and significance of new information” (information management strategies). This scale is widely used to examine adolescents’ and adults’ metacognitive awareness (e.g., Stringer and Looney, 2021; Güneş, 2022).

Instruments measuring metacognition in specific domains developed after Schraw and Dennison’s work (1994) often drew from the MAI. For instance, Zhang and Qin (2018) developed the Language Learners’ Metacognitive Writing Strategies in Multimedia Environment, assessing EFL learners’ writing metacognitive strategies used under the multimedia learning context. Sample items are “I planned what language features I was going to use in my essay with reference to the writing topic” and “I tried to seek help from an online dictionary if I did not know how to express my own opinions.” Drawing on the theoretical framework of metacognitive regulation strategies proposed by Wenden (1998), which includes planning, monitoring, and evaluating, it is also evident that Zhang and Qin’s (2018) Language Learners’ Metacognitive Writing Strategies in Multimedia Environment scale shares a significant resemblance to the MAI in terms of both the factor construct and the items design ideology. Hence, from the literature, it can be concluded that the MAI is a comprehensive and valid questionnaire assessing individuals’ general metacognitive knowledge and regulation. It can serve as guidance or a framework for designing domain-specific metacognitive scales. Nevertheless, most domain-specific metacognitive instruments focused on metacognitive strategies [e.g., the Language Learners’ Metacognitive Writing Strategies in Multimedia Environment scale by Zhang and Qin (2018); the Metacognitive Awareness of Reading Strategies Inventory by Mokhtari and Reichard (2002)]. In order to reflect individuals’ metacognitive knowledge and regulation of grit as a whole, the present research made an attempt and designed the Metacognitive Awareness of Grit Scale (MCAGS) drawing on the MAI (Schraw and Dennison, 1994).

Correspondingly, the design of MCAGS should also draw from the instruments measuring grit. In the study by Duckworth et al. (2007), where they coined the concept of grit with two constructs: perseverance of effort (POE) and consistency of interest (COI), they further developed a brief and stand-alone measurement that entails both of the constructs for adolescents and adults. A total of 12 items with six items for each construct consist of the grit scale, also known as the Grit-O scale. Sample items are “I become interested in new pursuits every few months” (COI) and “I finish whatever I begin” (POE). Nevertheless, Duckworth and Quinn (2009) later argued in their work that the Grit-O scale, although being a decent reflection of the two-factor structure proposed by Duckworth et al. (2007), failed to account for the differential predictive validity for various outcomes and the model fit of the Grit-O suggests room for improvement. Consequently, they further investigated the Grit-O scale and developed and validated the Short Grit Scale (Grit-S), a more efficient measurement of grit with better predictive validity than the Grit-O scale. The Grit-S scale only has nine items but still preserves the two-factor structure. As a valid and reliable measurement tool, the Grit-O and Grit-S (especially the Grit-S) scale was later used by many scholars in various domains (e.g., Tang et al., 2021; Zisman and Ganzach, 2021). Moreover, based on the structure of Grit-O and Grit-S, scholars have also designed domain-specific grit scales to address needs from particular research domains and contexts, such as the language domain-specific grit scale (L2-Grit) developed and validated by Teimouri et al. (2020).

### 2.4. The present research

Despite the fact that exploring the interaction between grit and metacognition is a novel area of research, empirical works from scholars have already begun to appreciate this interaction and its collective influence on academic achievement. For instance, Arslan et al. (2013) revealed that both constructs of grit positively correlated with learners’ metacognition. Furthermore, Wolters and Hussain (2015) reported that the student’s use of cognitive and metacognitive strategies serves as a successful mediator for the impact of grit on improved academic outcomes. Nevertheless, the extant empirical research in this domain mostly applied the general metacognitive awareness scales such as the Metacognitive Awareness Inventory (MAI; Schraw and Dennison, 1994) with the Grit-O (Duckworth et al., 2007) or the Grit-S (Duckworth and Quinn, 2009) scale. The advantages of the self-report questionnaire are time-saving, easy to administer to a substantial number of participants, and resource-saving. Nonetheless, many scholars have also criticized the validity of conducting questionnaire research, especially when administering the general scales for domain-specific matters (e.g., Allon et al., 1994; Song et al., 2021). For instance, to what degree will the general metacognitive scales measure individuals’ state of their metacognitive awareness in a specific context? In considering this, some scholars designed specific instruments measuring metacognition in particular contexts, as we mentioned previously. Hence, to address this concern and to contribute to further understanding of the English learning process, we developed and validated a questionnaire measuring learners’ metacognitive awareness of grit situated in the second language learning context.

## 3. Methodology

### 3.1. Instrument design

As a scale measuring learners’ metacognitive awareness of grit, the kernel of this measurement is to examine learners’ metacognitive knowledge and strategies related to learning grit. What do learners know about their current state of grit? What strategies do they propose to use to promote their grit levels? Hence, the construction of the sentences for the MCAGS items took examples from the MAI items (Schraw and Dennison, 1994). The MAI consists of 8 factors from two general components with 52 items. We decided that we had better preserve the sentence structures, and the connotations of the MAI items as the MAI is a well-developed and widely used metacognitive awareness instrument. For instance, an item such as “I understand my intellectual strengths and weaknesses” from the MAI measures learners’ declarative knowledge of their intellectual state. To reflect learners’ declarative knowledge of their current grit status instead of their general intellectual state, we modified this item to “I am aware of the level of my perseverance in learning English.” Similar rewording processes were performed for the rest of the sub-components of the MAI. Such as the procedural knowledge MAI item “I find myself using helpful learning strategies automatically” was altered to “I use helpful strategies to maintain perseverance in learning English.” Nevertheless, we deem that not all items from the MAI can be appropriately reworded for the new scale, such as the item “I am good at remembering information.” The item-wording based selection method is our primary item selection criterion as the original MAI is designed to reflect individuals’ metacognition based on their general cognitive processes, while the connotation of grit can be classified into the positive emotion category. The distinctions between MAI and grit signify that many MAI items are inappropriate when we attempted to convert them to reflect the learners’ metacognitive processes of maintaining and improving grit levels. Moreover, considering that items from the MAI need to represent the two factors of grit equally, the number of items in the MCAGS will be twice that of the selected MAI items. Considering the appropriateness of item-conversions and the expected size of the MCAGS, 24 items were selected from the MAI as examples representing the two-factor construct of *knowledge of cognition* and *regulation of cognition*, with each subcomponent also being addressed.

As mentioned earlier, the construct of the grit scales (Grit-O by Duckworth et al., 2007, and Grit-S by Duckworth and Quinn, 2009) entails two components, perseverance of effort (POE) and consistency of interest (COI). The items from these two factors either focus on learners’ ability to exert sustained effort by enumerating behaviors that bear relative traits, such as “I finish whatever I begin,” or examine learners’ capacity to maintain constant interests in the learning course by identifying actions that learners might do to distract their current focus (items were reverse scored to characterize constant interest), such as “I become interested in new pursuits every few months.” Since the essential function of the MCAGS is to measure learners’ metacognitive awareness, we decided to just extract the idea of POE and COI as two concepts instead of referring to the items of the grit scales and incorporate the grit factors into the two-factor structure of the MAI to form the MCAGS. Hence, a four-factor structure was created with 48 items (see the Supplementary material for the full questionnaire) comprising the factor of *knowledge of perseverance of effort* (KP; 9 items), *regulation of perseverance of effort* (RP; 15 items), *knowledge of consistency of interest* (KC; 9 items), and *regulation of consistency of interest* (RC; 15 items). For content validity, two experts with expertise in educational psychology, second language learning, metacognition, cognitive psychology, and positive psychology were invited to scrutinize the items and latent factors. Content that may induce confusion and grammatical errors was revised based on their suggestions. Regarding the language of the scales, we designed the bilingual scale with Chinese and English statements appearing together. To ensure the quality of both versions, the scale was reviewed by an expert specializing in Chinese-English and English-Chinese translation for the Chinese version. The English version was scrutinized by an expert in EFL whose native language is English. It is worth noting that English is a mandatory subject for all Chinese students from third year in primary school to university graduate programs. As part of the College Entrance Examination, students admitted into the Chinese University had already been equipped with sufficient English reading proficiency to comprehend the items designed in English. Nevertheless, we decided to distribute the scale in bilingual form. Each item was presented in both English and Chinese. In doing so, we provided the participants with the bilingual form where the two languages were used to ensure that the participants fully understood the connotation of the items in the scale.

### 3.2. Participants and procedures

A total of 859 Chinese student participants from a northern Chinese University were recruited and were asked to fill out the original 48-item Metacognitive Awareness of Grit Scale (MCAGS). Undertaking the present study in a Chinese university is meritorious, especially when the context is associated with learning English as a Foreign Language. As mentioned earlier, all domestic students in the Chinese universities must learn English throughout their entire educational experiences from primary to university, making them a perfect sample for EFL research. Additionally, as the present research concentrates on English language learners, we decided to include a wide range of student populations, including students in undergraduate, postgraduate and doctoral programs. With the large sample size, we believe the sample should be representative of the population than a selected small group of students, such as undergraduates. Aside from responding to the scale items, participants were also instructed to report their gender, age, and grade, with an average age of 21.83, ranging from 16 to 55. See Table 1 for the detailed report.

**Table 1:** Demographic information of the participants.

Category	*N*	*%*
Gender		
Male	412	48
Female	443	51.6
Others	4	0.5
Total	859	100
Age		
16–19	169	19.7
20–21	286	33.3
22–24	287	33.4
25–55	116	13.6
Total	858 (1 Missing)	100
Grade		
Freshman	172	20
Sophomore	246	28.6
Junior	212	24.7
Senior	110	12.8
First-year postgraduate study	36	4.2
Second-year postgraduate study	26	3
Third-year postgraduate study	15	1.7
First-year Ph.D. study	19	2.2
Second-year Ph.D. study	1	0.1
Third-year Ph.D. study	7	0.8
Fourth-year and above Ph.D. study	2	0.2
Others	13	1.5
Total	859	100

A convenience sampling strategy was applied. The initial MCAGS was imported into the Wenjuanxing platform, a sophisticated Chinese online platform that excels in questionnaire design and distribution. The link to the MCAGS generated by the platform was later shared with participants through WeChat. Prior to accessing the questionnaire, participants were presented with a consent form and a Participant Information Sheet, informing their rights of participation and withdrawal, and ensuring their anonymity. The present research and its materials were reviewed and approved by the University of Auckland Human Participants Ethics Committee. Participants were also instructed to respond to each item on a seven-point Likert scale (1. I strongly disagree; 2. I disagree; 3. I somewhat disagree; 4. Somewhere between agree and disagree; 5. I somewhat agree; 6. I agree; 7. I strongly agree.) Several concepts that may cause confusion were also explained, such as the notion of “perseverance.” The data collection procedure took 5 months.

## 4. Results

### 4.1. Descriptive statistics

Descriptive analysis found that item mean scores ranged from 4.47 to 5.31, with standard deviations ranging from 1.38 to 1.76. No missing values occurred.

### 4.2. Confirmatory factor analysis

**Figure 1:**
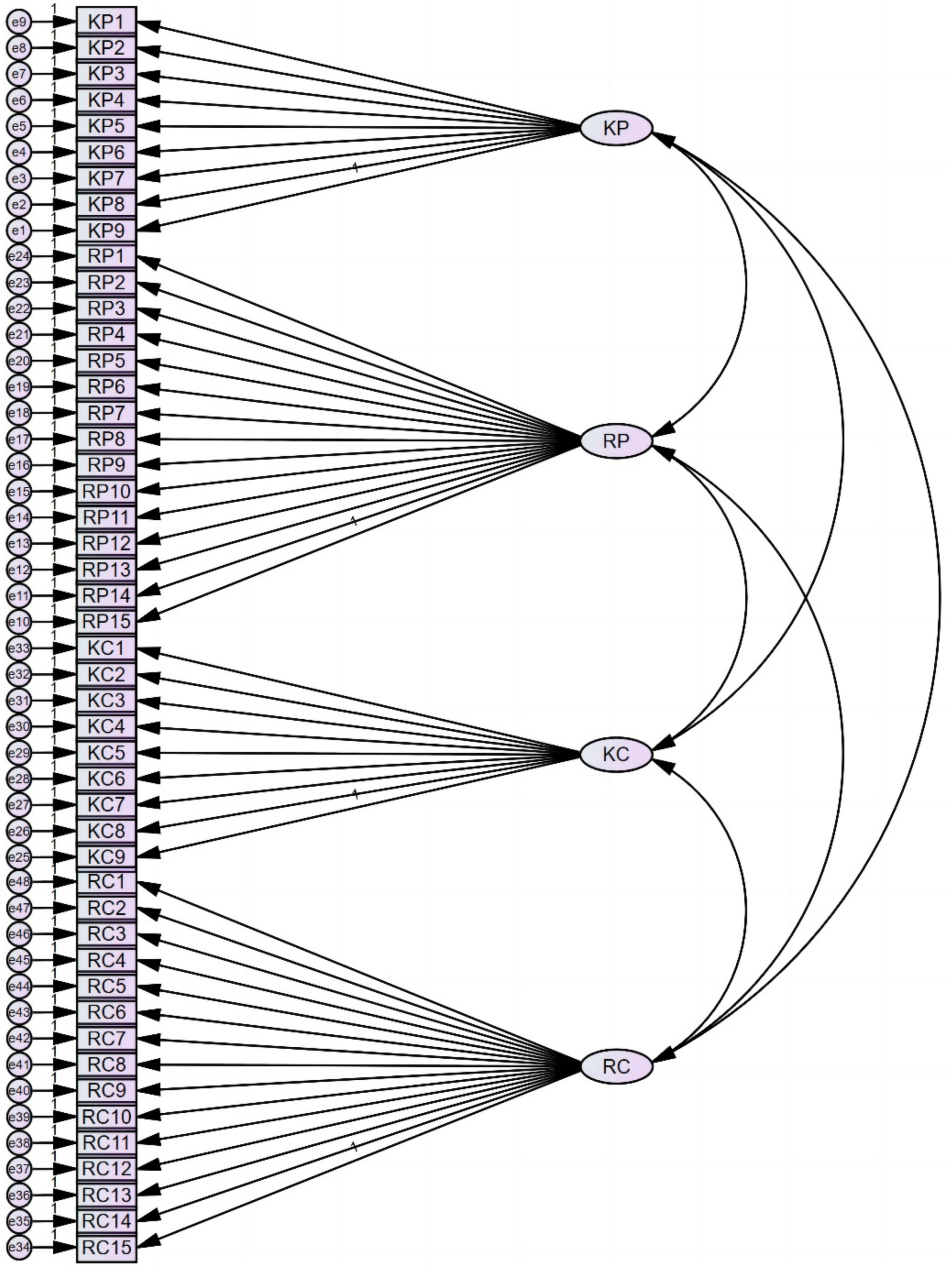
Initial model (48 items).

Confirmatory factor analysis (CFA) with Maximum Likelihood estimation was conducted using AMOS 26.0 to explore the factorial relationship between items and their corresponding latent factors. Considering that the factor structure of the MCAGS was built upon existing literature and the four-factor structure was established preceding the design of items, conducting an exploratory factor analysis to explore the latent factor structure is not necessary. Hence, we performed a confirmatory factor analysis to examine whether the items fit in the pre-determined factor structure. The initial model with 48 items is revealed in Figure 1. Factor loadings from CFA with the initial model yielded acceptable loadings for all items (Table 2).

**Table 2:** Factor loadings of initial model (48 items).

KP		RP		KC		RC	
KP1	0.617	RP1	0.716	KC1	0.720	RC1	0.772
KP2	0.689	RP2	0.776	KC2	0.743	RC2	0.763
KP3	0.753	RP3	0.775	KC3	0.755	RC3	0.738
KP4	0.750	RP4	0.681	KC4	0.744	RC4	0.744
KP5	0.783	RP5	0.680	KC5	0.798	RC5	0.760
KP6	0.755	RP6	0.729	KC6	0.766	RC6	0.768
KP7	0.735	RP7	0.793	KC7	0.754	RC7	0.662
KP8	0.740	RP8	0.731	KC8	0.719	RC8	0.727
KP9	0.740	RP9	0.770	KC9	0.756	RC9	0.765
		RP10	0.751			RC10	0.734
		RP11	0.747			RC11	0.746
		RP12	0.754			RC12	0.701
		RP13	0.671			RC13	0.717
		RP14	0.707			RC14	0.754
		RP15	0.761			RC15	0.783

KP stands for *knowledge of cognition of perseverance of effort*; RP stands for *regulation of cognition of perseverance of effort*; KC stands for *knowledge of cognition of consistency of interest*; RC stands for *regulation of cognition of consistency of interest*.

As a method of evaluating the statistical model based on the factor structure, CFA aims to provide statistical values to describe whether each item fits into its corresponding latent factor. Hence, several indices that serve the purpose of examining model fit were consulted as suggested by past literature, which is CMIN/*df* (or χ^2^/*df*; Kline, 2015), CFI (Comparative Fit Index; Bentler, 1990), NFI (Normed Fit Index; Bentler and Bonett, 1980), RMSEA (Root Mean Square Error of Approximation; Joreskog and Sorbom, 1981), SRMR (Standardized Root Mean Squared Residual; Maydeu-Olivares, 2017), GFI (Goodness of Fit Index; Joreskog and Sorbom, 1984), and AGFI (Adjust Goodness of Fit Index; Joreskog and Sorbom, 1984). It is commonly suggested that a good model fit should have indices of χ^2^/*df* ≤ 3.0, CFI ≥ 0.90, NFI ≥ 0.90, RMSEA ≤0.08, SRMR ≤0.10, GFI ≥ 0.90, and AGFI ≥0.90. Nevertheless, for the initial 48-item model, model fit indices from CFA suggest room for improvement (Table 3).

**Table 3:** Model fit indices of the initial model.

Indices	χ^2^/*df*	CFI	NFI	RMSEA	SRMR	GFI	AGFI
Model values	4.272	0.885	0.856	0.062^*^	0.039^*^	0.791	0.771
Cutoff criteria	≤3.0	≥0.90	≥0.90	≤0.08	≤0.10	≥0.90	≥0.90

^*^Is a model value that meets the suggested cutoff criteria. χ^2^ = 4588.619; *df* = 1,074.

**Figure 2:**
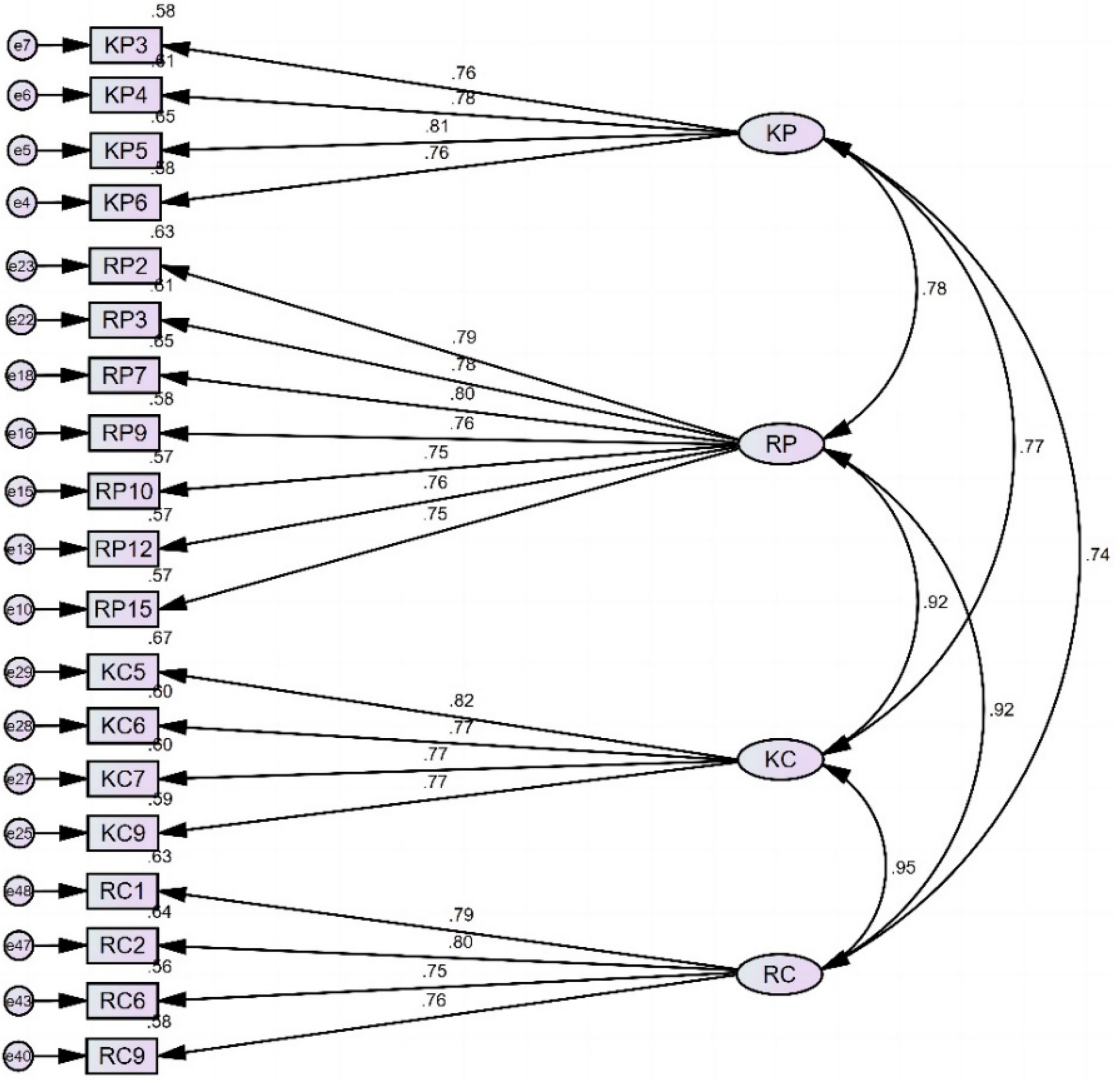
Model 2 (with standardized estimates).

With only two indices meeting the suggested criteria (RMSEA and SRMR), the initial model is insufficient to capture the relationship between items and factors. The initial model design allowed for sufficient redundancy to trim items based on factor loadings. Each connotation usually had several expressions (items). However, in viewing the high factor loadings for most items, following the common rule of thumb we thought that trimming items with factor loadings lower than 0.600 was not of any use, nor meaningful. Hence, we decided to remove items with factor loading smaller than 0.750, leaving a model (Model 2) that was still able to preserve the factor structure while improving the model fit. We further examined the items that were retained in model 2 and we deemed that these items were still able to capture the connotations of their corresponding factors. Consequently, 19 items remained for model 2 with factor loadings greater than 0.750 (Figure 2). CFA on model 2 with 19 items exhibits improved model fit indices (Table 4).

**Table 4:** Model fit indices of model 2.

Indices	χ^2^/*df*	CFI	NFI	RMSEA	SRMR	GFI	AGFI
Model values	3.664	0.965^*^	0.952^*^	0.056^*^	0.039^*^	0.938^*^	0.920^*^
Cutoff criteria	≤3.0	≥0.90	≥0.90	≤0.08	≤0.10	≥0.90	≥0.90

^*^Is the model value that meets the suggested cutoff criteria. χ^2^ = 534.873; *df* = 146.

**Figure 3:**
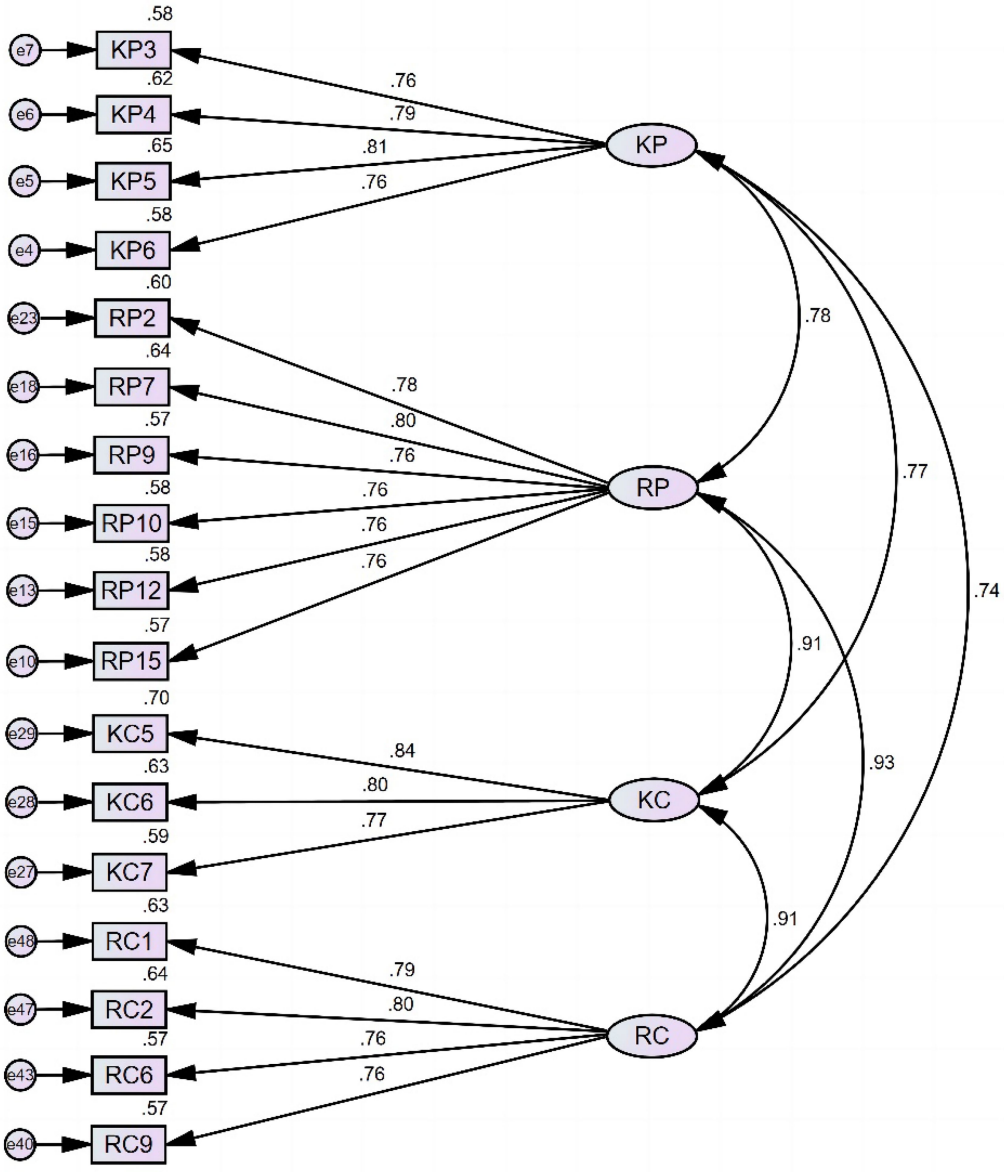
Model 3 with standardized estimates.

Albeit the fact that most of the model fit indices of model 2 fall within the acceptable criteria, the value of χ^2^/*df* is still problematic, suggesting further modification is required. Hence, we consulted the modification indices to seek opportunities to improve the model fit. The modification indices are values estimating the reduction of the chi-squared (χ^2^) value if a parameter restriction is lifted. In other words, the value of a modification index (MI) represents to what degree the model can be improved. The larger the value, the better improvement will be witnessed with the re-estimation. The standard technique is to create covariance between the errors that have large MI. One justification for creating correlations between errors is that items with large MI share a remarkable resemblance in terms of wording or connotation. However, such covariances can only be created between errors loaded into the same factor. For model 2, the MI for covariance between e22 and e23 is 36.908, 23.715 for covariance between e15 and e22, and 27.537 for covariance between e25 and e28. Theoretically, creating correlations between these errors should improve model fit. Nevertheless, researchers have cautioned against such practices, stating that such a data-driven model modification method rarely leads to an improved population model (MacCallum et al., 1992). Hence, we inspected the potentially problematic items instead of simply creating covariances between the errors. For instance, for e22 and e23 and the corresponding items, RP2 states, “Thinking about what I really need to do helps me persist in learning English,” and RP3 states, “It helps me persist in learning English if I organize my time.” Both of these statements intend to measure individuals’ regulation of cognition of perseverance of effort with the connotation of planning, and they share a remarkable resemblance in terms of wording. Moreover, “organize my time” can be viewed as a planning strategy that can be included in the “Thinking about what I really need to do” process. Hence, we decided to remove item RP3 as its connotation is implied by item RP2. As for e25 and e28 and the corresponding items, KC6 states, “I use strategies to maintain my interest in learning English,” and KC9 states, “My purposes help me keep a constant interest in learning English.” Similarly, setting purposes can also be regarded as using strategies. For this pair, we decided to preserve item KC6, as measuring the behavior of using strategies to maintain interest in learning English is closer to our intention of exploring individuals’ use of metacognitive strategies. At this point, a 17-item model was preserved (model 3). CFA was performed again to examine the model fit. Figure 3 exhibits the model 3 diagram, and Table 5 reports the model fit indices.

**Table 5:** Model fit indices of model 3.

Indices	χ^2^/*df*	CFI	NFI	RMSEA	SRMR	GFI	AGFI
Model Values	2.843^*^	0.978^*^	0.966^*^	0.046^*^	0.025^*^	0.959^*^	0.944^*^
Cutoff Criteria	≤3.0	≥0.90	≥0.90	≤0.08	≤0.10	≥0.90	≥0.90

^*^Is the model value that meets the suggested cutoff criteria. χ^2^ = 321.310; *df* = 113.

As can be seen in Table 5, model 3 with 17 items witnessed an improvement of model fit for all indices, and the values fall within the recommended cutoff criteria. Hence, a 17-item Metacognitive Awareness of Grit Scale (MCAGS) was preserved as the preferred model (see Table 6 for the item list).

**Table 6:** Seventeen-item MCAGS.

Metacognitive Awareness of Grit Scale	
Factor 1	Knowledge of cognition of perseverance of effort (KP)
Items	I am a good judge of how well I can persist in learning English. 我对我能够在多大程度上坚持学习英语有着良好的判断。
	I have control over my perseverance in learning English. 我能够控制自己保持学习英语的毅力。
	I use strategies that have worked in the past to help me maintain perseverance in learning English. 我会使用过去行之有效的策略和方法来帮助我保持学习英语的毅力。
	I use helpful strategies to maintain perseverance in learning English. 我会使用一些策略和方法来帮助我保持学习英语的毅力。
Factor 2	Regulation of Cognition of Perseverance of Effort (RP)
Items	Thinking about what I really need to do helps me persist in learning English. 仔细考虑我真正要做的事对于我坚持学习英语有帮助。
	Constantly learning new information helps me persist in learning English. 不断地学习新知识能够帮助我坚持学习英语。
	Breaking down studying into smaller steps helps me persist in learning English. 将学习任务细化成小的步骤能够帮助我坚持学习英语。
	I re-evaluate the strategies of effort if I am about to give up. 在我即将放弃的时候, 重新评估我所使用的策略和方法能够帮助继续坚持学习英语。
	I ask others for advice on how to persist in learning English. 我会向其他人请教能够帮助我坚持学习英语的策略和方法。
	I evaluate if I put to good use strategies that can help me persist in learning English. 我会问我自己是否充分利用了能够帮助我坚持学习英语的策略和方法。
Factor 3	Knowledge of Cognition of Consistency of Interest (KC)
Items	I use strategies that have worked in the past to help me maintain my interest in learning English. 我使用过去行之有效的方法和策略来帮助我保持学习英语的兴趣。
	I use strategies to maintain my interest in learning English. 我会自发的使用有效的方法和策略来帮助我保持学习英语的兴趣。
	I motivate myself to maintain interest in learning English when I need to. 在我需要的时候, 我可以激励我自己来保持学习英语的兴趣。
Factor 4	Regulation of Cognition of Consistency of Interest (RC)
Items	Setting specific goals helps me maintain a consistent interest in learning English. 设立详细的学习目标能够帮助我保持学习英语的兴趣。
	I think about what I really need to do to help me maintain my interest in learning English. 我会认真考虑我真正需要做的事情, 以此来帮助我保持学习英语的兴趣。
	I analyze the usefulness of strategies that help me maintain a constant interest in learning English. 我会分析我所使用的策略和方法来探究其是否能够帮助我保持学习英语的兴趣。
	Breaking down studying into smaller steps helps me maintain a constant interest in learning English. 将学习目标分解成几个步骤能够帮助我保持学习英语的兴趣。

A principal components analysis with Promax rotations using SPSS 27.0 was conducted to examine the final model variances. Factors from the final model together explained 70.412% of the variance and are reliable measurements based on Cronbach α scores (Table 7).

**Table 7:** Factor variances and reliability tests.

	Factor variances explained (%)	Reliability (*α*)
Factor 1	7.020	0.860
Factor 2	56.073	0.897
Factor 3	3.488	0.842
Factor 4	3.832	0.859
Full model	70.412	0.951

### 4.3. Hierarchical confirmatory factor analysis

In spite of the fact that the factor structure of model 3 exhibits excellent model fit, a problem emerges in terms of the scale’s discriminant validity. It is evident from Figure 3 that all four factors are highly correlated with each other. Hence, a potential higher-order factor structure that can explain the correlations is suggested. Considering that the four-factor structure is built upon the construct of metacognition and grit, each of these two constructs has two subcomponents as discussed in previous sections (knowledge of cognition and regulation of cognition for metacognition, and perseverance of effort and consistency of interest for grit). We then identified these two constructs as two second-order factors and conducted the hierarchical confirmatory factor analysis (HCFA) separately to see if the model fit can be significantly improved by adding a higher-order factor into the model.

#### 4.3.1. Hierarchical confirmatory factor analysis with metacognition as the second-order factor (model 4)

**Figure 4:**
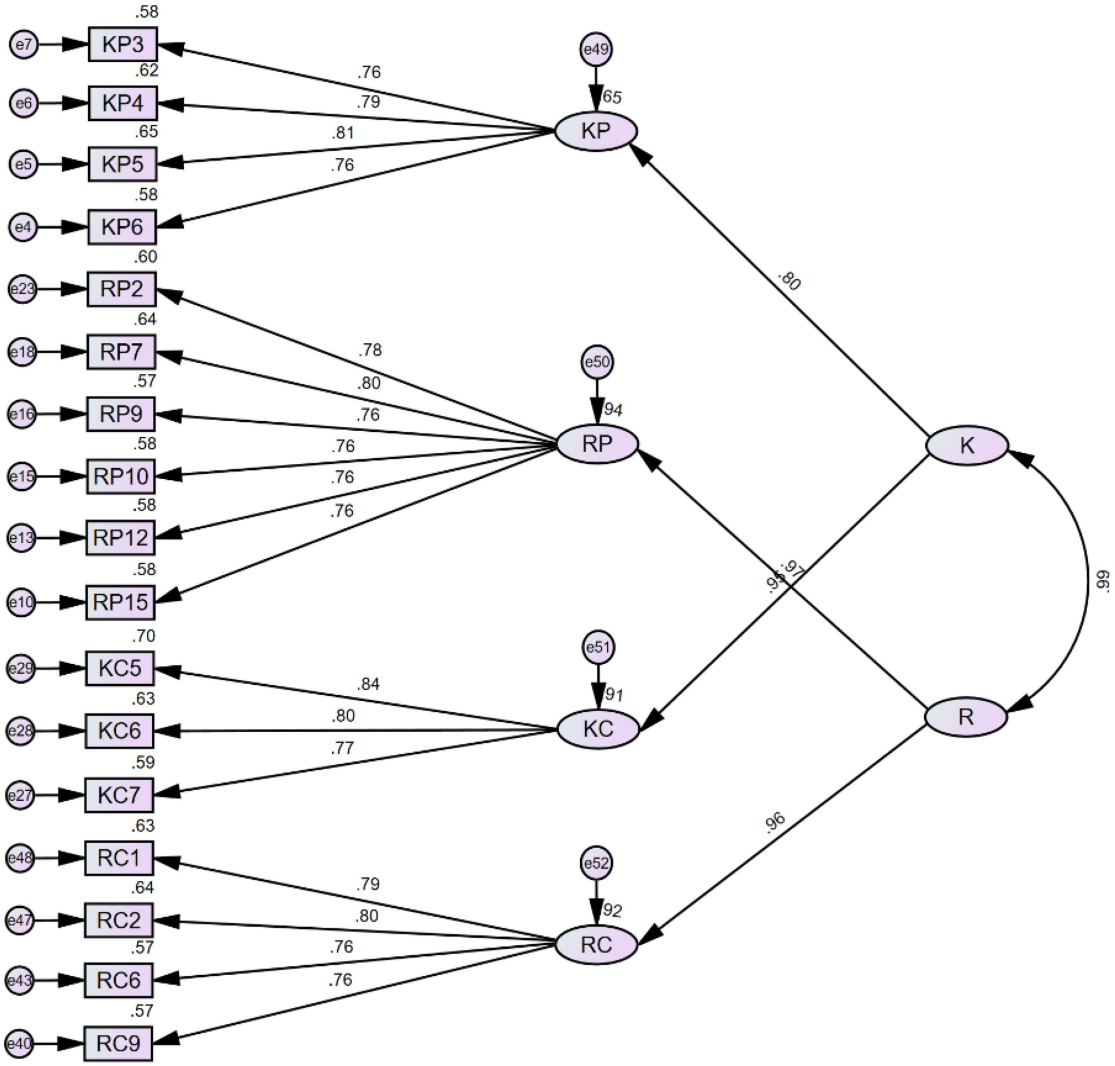
Model 4 with metacognition as the second-order factor. *K* stands for *knowledge of cognition* and *R* stands for *regulation of cognition.*

The two constructs of metacognition were added to the model as the second-order factor (see Figure 4). KP and KC were loaded to the factor of *knowledge of cognition*, whereas RP and RC were loaded to the factor of *Regulation of cognition*. The HCFA results suggest that Model 4 is still an excellent fit (see Table 8 for model fit indices).

**Table 8:** Model fit indices of model 4.

Indices	χ^2^/*df*	CFI	NFI	RMSEA	SRMR	GFI	AGFI
Model values	2.865^*^	0.978^*^	0.966^*^	0.047^*^	0.026^*^	0.958^*^	0.944^*^
Cutoff criteria	≤3.0	≥0.90	≥0.90	≤0.08	≤0.10	≥0.90	≥0.90

^*^Is the model value that meets the suggested cutoff criteria. χ^2^ = 326.599; *df* = 114.

However, it is worth noting that the *χ*^2^ value of model 4 is larger than that of model 3 (*χ*^2^ = 321.310; *df* = 113). It implies that model 4, with metacognition as the second-order factor, potentially fits the model less ideally than model 3. We further investigated the *χ*^2^ difference by computing χ^2^_M4_-χ^2^_M3_ (5.289) and *df*_M4_-*df*_M3_ (1). The *value of p* for this difference is 0.021. To this point, we can conclude that by including a higher-order factor representing the construct of metacognition (Knowledge of cognition and regulation of cognition), the new model is significantly poorer than the original model that only contains the first-order factor structure.

#### 4.3.2. Hierarchical confirmatory factor analysis with grit as the second-order factor (model 5)

**Figure 5:**
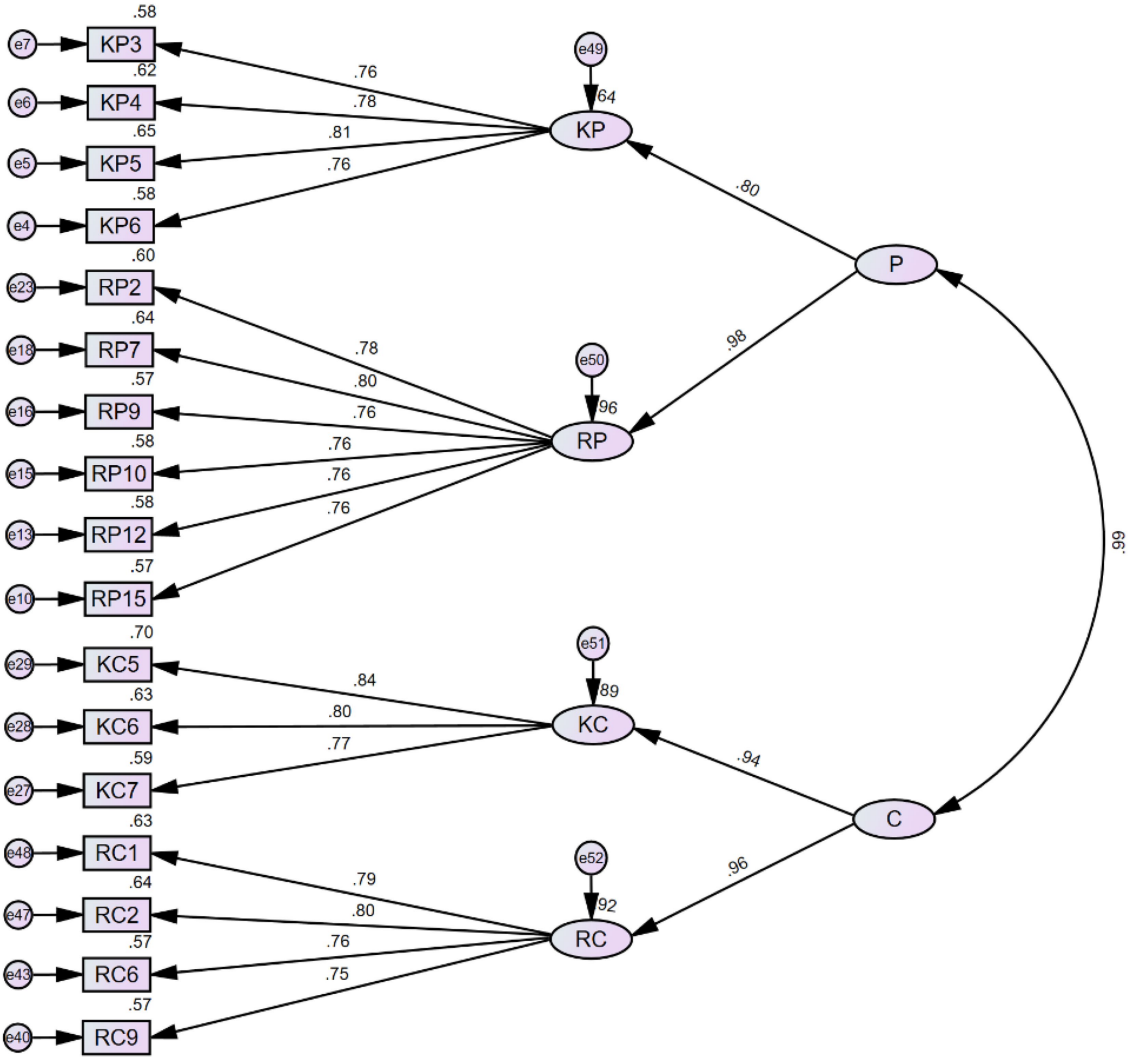
Model 5 with grit as the second-order factor. P stands for *perseverance of effort* and C stands for *consistency of interest.*

In a similar fashion, we also explored the model fit when incorporating grit into the model as the second-order factor (see Figure 5). KP and RP were loaded to the *perseverance of effort*, whereas KC and RC were loaded to the *consistency of interest*. The HCFA results suggest that Model 5 is also an excellent fit (see Table 9 for model fit indices).

**Table 9:** Model fit indices of model 5.

Indices	χ^2^/*df*	CFI	NFI	RMSEA	SRMR	GFI	AGFI
Model values	2.862^*^	0.978^*^	0.966^*^	0.047^*^	0.026^*^	0.958^*^	0.944^*^
Cutoff criteria	≤3.0	≥0.90	≥0.90	≤0.08	≤0.10	≥0.90	≥0.90

^*^Is the model value that meets the suggested cutoff criteria. χ^2^ = 326.229; *df* = 114.

Not surprisingly, the *χ*^2^ value of model 5 is also larger than that of model 3 (*χ*^2^ = 321.310; *df* = 113). The *χ*^2^ difference between model 5 and model 3 is also computed with a *p-*value (χ^2^_M5_-χ^2^_M3_ = 4.919; *df*_M5_-*df*_M3_ = 1; *p* = 0.027). It is apparent that the significant *value of p* represents substantial damage to the model fit after including the two-factor construct of grit as a higher-order factor in the CFA when compared with model 3.

#### 4.3.3. Hierarchical confirmatory factor analysis with a single factor as the second-order factor (model 6)

**Figure 6:**
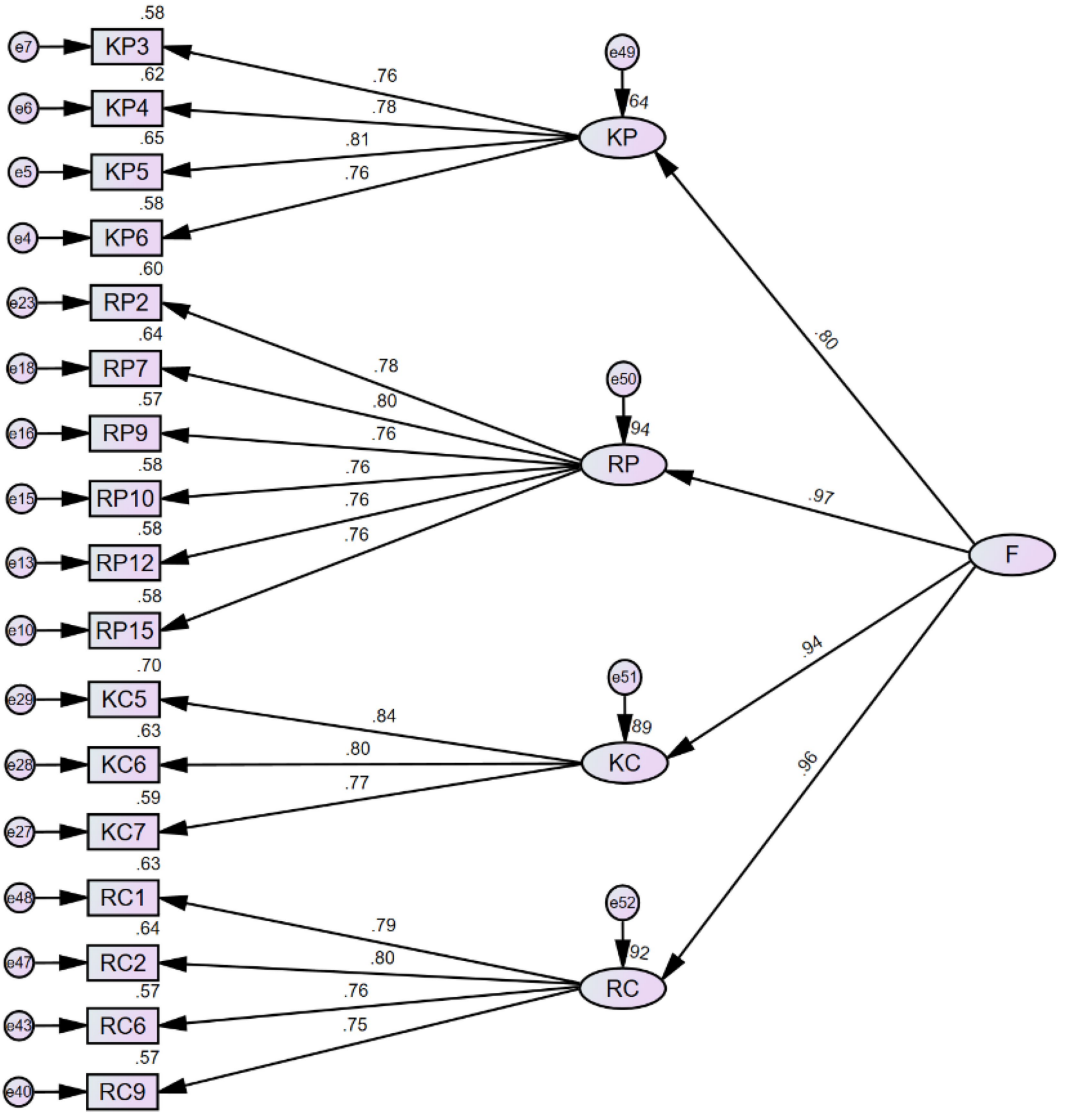
Model 6 with a single factor as the second-order factor. *F* stands for the single factor that we proposed.

It is self-evident from Figures 4, 5 that the correlation between the two factors of the second-order structure is also very high, which leads to a further assumption that the four factors in the first-order structure can be grouped into a single factor as the higher-order factor. There is no theoretical background to support such a grouping strategy, as the constructs of metacognition and grit are distinct in many ways. Nevertheless, we still attempted to test whether a single factor serving as the higher-order factor can significantly improve the model fit (see Figure 6 for Model 6 and Table 10 for model fit indices).

**Table 10:** Model fit indices of model 6.

Indices	χ^2^/*df*	CFI	NFI	RMSEA	SRMR	GFI	AGFI
Model values	2.852^*^	0.977^*^	0.966^*^	0.046^*^	0.026^*^	0.958^*^	0.944^*^
Cutoff criteria	≤3.0	≥0.90	≥0.90	≤0.08	≤0.10	≥0.90	≥0.90

*Is the model value that meets the suggested cutoff criteria. χ^2^ = 328.022; *df* = 115.

Similarly, the *χ*^2^ value of model 6 is also larger than model 3 (*χ*^2^ = 321.310; *df* = 113). The *χ*^2^ difference between model 6 and model 3 is also computed with a *value of p* (χ^2^_M6_-χ^2^_M3_ = 6.712; *df*_M6_-*df*_M3_ = 2; *p* = 0.035). To this point, the result illustrated that adding a single factor as the second-order factor into the model will not improve the model fit.

In sum, the exploration of HCFA did not offer us new insights pertaining to the relationship among the four factors of the model. Contrary to our anticipation, adding a possible second-order factor significantly damaged the model fit. Consequently, model 3 is retained as the final Metacognitive Awareness of Grit Scale (MCAGS), which consists of 17 items, covering *Knowledge of cognition of Perseverance of effort* (four items: Item KP3, 4, 5, 6), *Regulation of cognition of Perseverance of effort* (six items: Item RP2, 7, 9, 10, 12, 15), *Knowledge of cognition of Consistency of interest* (three items: Item KC5, 6, 7), and *Regulation of cognition of Consistency of interest* (four items: Item RC1, 2, 6, 9).

## 5. Discussion

This study is an attempt to develop and validate a scale measuring individuals’ metacognitive awareness of grit. To construct the factor structure of the Metacognitive Awareness of Grit Scale (MCAGS), we relied on the items and factors design ideology of the Metacognitive Awareness Inventory (MAI) by Schraw and Dennison (1994) while incorporating the concept of grit. Consequently, a four-factor MCAGS consists of 48 items was later reduced to 17 as the final model.

### 5.1. The MCAGS

The first factor (*Knowledge of Cognition of Perseverance of Effort*) focuses on individuals’ evaluation of their own judgment of their capabilities to exert consistent effort in learning English and their awareness of existing strategies to aid this process. In other words, different from assessing individuals’ states of their perseverance of effort (e.g., Grit-S), the first factor of the MCAGS represents learners’ reflections upon whether they have an apt judgment of perseverance of effort in learning English (item KP3), their own control of perseverance in English learning (item KP4), the efficacy of past helpful strategies (item KP5), and the actions of using helpful strategies (KP6). Simply put, the self-awareness of the capabilities to exert constant effort is examined.

The second factor (*Regulation of Cognition of Perseverance of Effort*) generally involves regulating learners’ current status of perseverance of effort. It implies that actions pertinent to the application of regulation strategies are beneficial for learners to persist in learning English. This factor examines learners’ strategies of planning the learning process (item RP2), constantly pursuing new knowledge (RP7), managing new information by breaking down materials (RP9), re-evaluating the usefulness of current strategies (RP10), solving potential problems (RP12), and re-evaluating their own performance of executing strategies to maintain perseverance in learning English. It is worth noting that factor two stresses the specific strategies used for regulation purposes to achieve perseverance in learning efforts. In contrast, factor one focuses only on the learners’ awareness of the existence of metacognitive strategies.

As for the third factor (*Knowledge of Cognition of Consistency of Interest*), similar to factor one, it examines learners’ awareness of the strategies to help them maintain consistent interest in learning English. Unlike the concept of engagement and enjoyment, consistency of interest stresses the capability to exhibit constant interest instead of momentary or short-term excitement in learning. Practical strategies also exist to aid learners in achieving this goal. Factor three serves to explore learners’ metacognitive knowledge regarding these strategies by focusing on learners’ attitudes toward applying strategies (KC6), procedural knowledge of using strategies (KC5), and proactive actions of motivating themselves to use helpful strategies to maintain consistent interest in learning English (KC7).

The final factor (*Regulation of Cognition of Consistency of Interest*) serves the same function as factor two, albeit the replacement of perseverance of effort with the consistency of interest. Strategies also exist to aid learners in maintaining a constant interest in English learning and thus are tested by factor four to see if individuals actually prefer to apply these strategies. More specifically, item RC1 enquires about learners’ goal-setting strategy for maintaining consistent interest in English learning, item RC2 examines the planning process, item RC6 implies the process of monitoring the strategy use, and item RC9 (same as RP9) also evaluates learners’ information management strategy.

The development of the MCAGS responds to the urgent need for a specifically designed instrument assessing individuals’ metacognitive knowledge and regulation of learning grit. It resonates with the consensus that metacognition and grit both serve as potent predictors of academic achievements (Duckworth and Quinn, 2009), which is the core argument of discussing the impact of metacognition and grit. Nevertheless, research tapping directly into the correlation between grit and metacognition is scant, albeit some discussions have been made, such as the work by Arslan et al. (2013) mentioned earlier. Additionally, the interaction between grit and self-regulated learning (a multi-faceted structure that embraces cognition, metacognition, motivational beliefs, and social behavior; Zimmerman, 2011) has recently gained attention from scholars. For instance, grit is significantly linked to self-regulation for college students (Pasha-Zaidi et al., 2019). Grit and both of its sub-components positively correlated with self-regulated learning strategies in the study by Martin et al. (2022). Perseverance of effort was a consistent and adaptive predictor for metacognitive strategies in the self-regulated learning framework (Wolters and Hussain, 2015). Hence, it should be stressed that research revolving around grit and metacognition is a novel research path and significantly influences learners’ learning performance and outcomes, yielding the desideratum for further exploration.

### 5.2. Implications, future directions, and limitations

Developing the MCAGS is a proactive effort to offer future researchers a domain-specific scale that can detect learners’ metacognitive knowledge and strategies to maintain constant effort and interest in learning English. As introduced in the earlier section, the application of domain-specific scales for specific purposes instead of general scales is encouraged by scholars (e.g., Song et al., 2021), stating that the generic nature of general instruments may fail to capture specific psychological constructs when participants respond to the instruments. For instance, the meta-analytical review by Ohtani and Hisasaka (2018) attributed a biased result to the possible cause of domain representativeness issue, arguing that the larger effect sizes of online metacognitive measurement methods compared with off-line methods are the results of the fact that off-line methods reflect domain-general metacognition while on-line methods could reflect domain-specific metacognition. Hence, the design of the MCAGS contributes to the effort to develop domain-specific metacognitive instruments.

The development of MCAGS opens up the potential for numerous future research opportunities to probe into the realm of metacognition and grit. The application of the MCAGS could be meaningful in exploring the impact of learners’ metacognitive awareness of grit on academic achievement, motivation, self-efficacy, and even the concept of grit itself. Considering that the MCAGS predominantly assess learners’ evaluation of their own actions of assessing and executing metacognitive strategies to maintain constant effort and interest in English learning, it is justifiable to presume that the score of MCAGS should positively correlate with learners’ grit level. Furthermore, it is also reasonable to hypothesize that learners with a high score on the MCAGS should witness elevated academic performance and improved self-efficacy. From a more general perspective, most of the constructs associated with grit bear promising future research significance when connected with the MCAGS.

Although the design of the MCAGS put a heavy value on the metacognitive aspects and composed the factors and items based on the MAI, the application of the MCAGS should primarily focus on its assessment of grit. Hence, probing the relationship between the MCAGS and other positive psychological constructs may also yields promising results. Moreover, it is also critical for future researchers to extend the context of the MCAGS to other domains of education and psychology. With a small amount of tuning of the items, the MCAGS should also be applicable to learning contexts other than English. As a trait-based personality construct (Wang, 2021), grit (and the MCAGS) should not be sensitive to the changes in ethnicity and culture in terms of assessment validity and reliability, while the score of the MCAGS and its relationship with other constructs might differ, which is also an exciting breakthrough point for future research.

The development of the MCAGS is not perfect in several ways. Firstly, the wording of the items, although exhibiting acceptable performance regarding statistical results (e.g., the modification indices), share a certain level of resemblance with each other. Future research may exert effort to modify some of the items. Secondly, the MCAGS poses a discriminant validity issue. The four constructs are not unrelated, especially between RP and KC, RP and RC, and KC and RC. One possible explanation for this unexpected result is that the design of the MCAGS constructs is the result of the interaction between the constructs of the MAI and the Grit measurements. Nonetheless, the HCFA results did not reveal an improved model fit. Future research should take this into account while applying the MCAGS.

## 6. Conclusion

The present research offered future researchers a domain-specific instrument measuring English learners’ metacognitive awareness of grit. Such an endeavor can be viewed as a response to the need to create domain-specific instruments. Hence, it both presents academic implications for filling the lacuna of this realm and bears significant practical application for various research domains. Designing new domain-specific instruments deserves more attention as they are more robust measurements than domain-general instruments when addressing specific issues. Moreover, developing new instruments also signifies more possibilities for future research, which should greatly expand the current research domain and encourage researchers to produce more intellectual achievements. Finally, designing an instrument in a bilingual form is advantageous as it gives participants a better chance to understand the items fully. Such merit could be more prominent for EFL research in a non-English cultural context.

## Data availability statement

The original contributions presented in the study are included in the article/Supplementary material, further inquiries can be directed to the corresponding author.

## Ethics statement

The studies involving human participants were reviewed and approved by the University of Auckland Human Ethics Committee. The patients/participants provided their written informed consent to participate in this study.

## Author contributions

MW and LZ conceived and designed the study, with RH’s support. MW collected and analyzed the data and drafted the manuscript. LZ finalized it for submissions as the corresponding author. All the authors revised and approved the manuscript.

## Conflict of interest

The authors declare that the research was conducted in the absence of any commercial or financial relationships that could be construed as a potential conflict of interest.

## Publisher’s note

All claims expressed in this article are solely those of the authors and do not necessarily represent those of their affiliated organizations, or those of the publisher, the editors and the reviewers. Any product that may be evaluated in this article, or claim that may be made by its manufacturer, is not guaranteed or endorsed by the publisher.
