# Supplementary material for: Developing the Metacognitive Awareness of Grit Scale for a better understanding of learners of English as a foreign language
Source: Front Psychol. 2023 Apr 11;14:1141214. doi: 10.3389/fpsyg.2023.1141214 (PMC10126521; doi:10.3389/fpsyg.2023.1141214)
Supplement: Supplementary file 1 [file Data_Sheet_1.pdf]

Metacognitive Awareness of Grit Scale (48-item original scale)

This scale should be answered on a seven-point Likert Scale.
1- I strongly disagree
我非常不同意
2- I disagree
我不同意
3- I somewhat disagree
我有点不同意
4- Somewhere between agree and disagree
我不确定
5- I somewhat agree
我有点同意
6- I agree
我同意
7- I strongly agree
我非常同意

No reverse scoring item is designed.

Dimension one: Knowledge of Cognition of Perseverance of Effort (KP)

1. I am aware of the level of my perseverance in learning English.
   我能够认识到我在学习英语上有多大的毅力。
2. I am aware of the strategies I can use to help me persist in learning English.
   我知道有许多方法和策略供我使用来帮助我坚持学习英语。
3. I am a good judge of how well I can persist in learning English.
   我对我能够在多大程度上坚持学习英语有着良好的判断。
4. I have control over my perseverance in learning English
   我能够控制自己保持学习英语的毅力。
5. I use strategies that have worked in the past to help me maintain perseverance in learning English.
   我会使用过去行之有效的策略和方法来帮助我保持学习英语的毅力。
6. I use helpful strategies to maintain perseverance in learning English.
   我会使用一些策略和方法来帮助我保持学习英语的毅力。
7. I motivate myself to persevere in learning English.
   如果我需要的话，我会鼓励我自己来坚持学习英语。
8. I motivate myself to persevere when I know how to learn English.
   如果我知道如何学习英语，我就能鼓励我自己坚持学习英语。
9. My purposes help me persist in learning English.
   树立学习英语的目的使我能够坚持学习英语。

Dimension two: Regulation of Cognition of Perseverance of Effort (RP)

10. Setting specific goals helps me persist in learning English.

制定详细的学习目标对于我坚持学习英语有帮助。

11. Thinking about what I really need to do helps me persist in learning English.
仔细考虑我真正要做的事对于我坚持学习英语有帮助。

12. It helps me persist in learning English if I organize my time.
规划我的学习时间对于我坚持学习英语有帮助。

13. I ask myself if I am still consistently devoting effort to learning English.
我时常问自己是否还在坚持学习英语。

14. I ask myself if I have done all I can to help me persist in learning English.
我会问自己是否做到了竭尽所能坚持学习英语。

15. I analyze the usefulness of strategies that helped me persist in learning English.
我会分析帮助我坚持学习英语的策略的有效性。

16. Constantly learning new information helps me persist in learning English.
不断地学习新知识能够帮助我坚持学习英语。

17. Translating knowledge into my own words helps me persevere in learning English.
将学习到的知识转化成我自己的理解能够帮助我坚持学习英语。

18. Breaking down studying into smaller steps helps me persist in learning English.
将学习任务细化成小的步骤能够帮助我坚持学习英语。

19. I re-evaluate the strategies of effort if I am about to give up.
在我即将放弃的时候，重新评估我所使用的策略和方法能够帮助继续坚持学习英语。

20. I change strategies when I cannot persist in learning English.
当我无法继续坚持学习英语时，我会尝试改变策略和方法来继续坚持下去。

21. I ask others for advice on how to persist in learning English.
我会向其他人请教能够帮助我坚持学习英语的策略和方法。

22. I consistently evaluate my level of perseverance in learning English.
我会经常评估我现在的英语学习毅力程度。

23. I evaluate whether some strategies are better than others to help me persist in learning English.
我会问我自己是否还有更好的策略和方法来帮助我坚持学习英语。

24. I evaluate if I put to good use strategies that can help me persist in learning English.
我会问我自己是否充分利用了能够帮助我坚持学习英语的策略和方法。

Dimension three: Knowledge of Cognition of Consistency of Interest (KC)

25. I am aware of the consistency of my level of interest in learning English.
我知道我能多大程度长时间保持对英语学习的兴趣。

26. I am a good judge of how well I can maintain a consistent interest in learning English.
我对我能够在多大程度上保持对学习英语的兴趣有着良好的判断。

27. I know that there are strategies I can use to help me maintain my interest in learning English.
我知道我可以使用方法和策略来帮助我保持对英语学习的兴趣。

28. I have control over maintaining a consistent interest in learning English
我能够控制自己对英语学习保持持续的兴趣。

29. I use strategies that have worked in the past to help me maintain my interest in learning English.

我使用过去行之有效的方法和策略来帮助我保持学习英语的兴趣。

30. I use strategies to maintain my interest in learning English.
我会自发的使用有效的方法和策略来帮助我保持学习英语的兴趣。

31. I motivate myself to maintain interest in learning English when I need to.
在我需要的时候，我可以激励我自己来保持学习英语的兴趣。

32. I motivate myself to maintain a consistent interest in learning English when I know how to learn it.
当我知道如何学习英语时，我就能鼓励我自己保持学习英语的兴趣。

33. My purposes help me keep a constant interest in learning English.
树立学习英语的目的有助于我保持对英语学习的兴趣。

Dimension four: Regulation of Cognition of Consistency of Interest (RC)

34. Setting specific goals helps me maintain a consistent interest in learning English.
设立详细的学习目标能够帮助我保持学习英语的兴趣。

35. I think about what I really need to do to help me maintain my interest in learning English.
我会认真考虑我真正需要做的事情，以此来帮助我保持学习英语的兴趣。

36. Organizing my time to study English helps me keep a constant interest in learning English.
合理规划我的学习时间能够帮助我保持学习英语的兴趣。

37. I ask myself periodically if I am still consistently interested in learning English.
我时常会问我自己是否对学习英语还抱有持续的兴趣。

38. I ask myself if I have done all I can to help me keep my interest in learning English.
我会问我自己是否做了我所有能做的事情来帮助我保持学习英语的兴趣。

39. I analyze the usefulness of strategies that help me maintain a constant interest in learning English.
我会分析我所使用的策略和方法来探究其是否能够帮助我保持学习英语的兴趣。

40. Constantly learning new information helps keep me interested in learning English.
不断地学习新知识能够帮助我保持学习英语的兴趣。

41. Translating knowledge into my own words while learning English is helpful for maintaining my constant interest in English learning.
将学到的知识转化为我自己的理解能够帮助我保持学习英语的兴趣。

42. Breaking down studying into smaller steps helps me maintain a constant interest in learning English.
将学习目标分解成几个步骤能够帮助我保持学习英语的兴趣。

43. I explore new possibilities instead of giving up if I cannot keep a constant interest in learning English.
如果我无法再保持对学习英语的兴趣，我会寻找新的方法和策略而不是放弃。

44. I re-evaluate my strategies to find out if they can still help me maintain a constant interest in learning English.
我会重新评估我正在使用的方法和策略，看它们是否还能帮助我保持学习英语的兴趣。

45. I ask others for advice on possible strategies to help me maintain a constant interest in learning English.
我会向其他人请教能够帮助我保持英语学习兴趣的策略和方法。

46. I consistently evaluate my level of interest in learning English.

我每隔一段时间都会评估我目前对于学习英语的兴趣水平。

47. I ask myself if there are better strategies to help me constantly be interested in learning English.

我会问我自己是否还有更好的策略和方法来帮助我保持对学习英语的兴趣。

48. I ask myself if I have put the strategies to good use that can help me maintain a constant interest in learning English.

我会问我自己是否充分利用了能够帮助我保持英语学习兴趣的策略和方法。
